# YouTube recommendations for indications and surgical decision-making in arthroscopic rotator cuff repair show low concordance with 2025 American Academy of Orthopaedic Surgeons Clinical Practice Guidelines

**DOI:** 10.1016/j.xrrt.2026.100837

**Published:** 2026-08-05

**Authors:** Shankar S. Thiru, Matthew Joy, Gabriel I. Onor, Kory BD. Pasko, Kerstin Yu, Bryan Murtaugh, Edward S. Chang, Dean Wang

**Affiliations:** a Department of Orthopaedic Surgery, University of California Irvine, Irvina, CA, USA; b Georgetown University School of Medicine, Washington, DC, USA; c Thomas Jefferson University Hospital, Rothman Orthopaedic Institute, Philadelphia, PA, USA; d MedStar National Rehabilitation Network, Washington, DC, USA; e Inova Sports Medicine, Fairfax, VA, USA

**Keywords:** Rotator cuff repair, Arthroscopic rotator cuff surgery, AAOS Clinical Practice Guidelines, YouTube, Surgical decision-making, Guideline concordance, Online health education

## Abstract

**Background:**

To evaluate the concordance of YouTube videos addressing rotator cuff tear management with the 2025 American Academy of Orthopaedic Surgeons (AAOS) Clinical Practice Guideline (CPG), with particular focus on treatment indications and surgical decision-making for arthroscopic rotator cuff repair, and to determine whether video characteristics are associated with guideline concordance.

**Methods:**

A cross-sectional content analysis of publicly available YouTube videos was performed between January and February 2026 using predefined search terms related to rotator cuff tear treatment and surgical decision-making. English-language videos ≥1 minute in duration discussing management of rotator cuff tears were included. Videos focused exclusively on post-operative rehabilitation were excluded. Two independent reviewers coded each video against 9 AAOS CPG-derived decision points, classifying each as concordant, discordant, or not addressed. Multivariable logistic regression was used to assess associations between video characteristics and above-median concordance.

**Results:**

A total of 108 videos were included. Median guideline concordance was 11.1% (interquartile range: 0.0-33.3). Twenty-four (22.2%) of videos addressed no decision-making domain. Highest concordance rates were observed for physical therapy effectiveness (45.4%), surgical indications (46.3%), and management of high-grade partial-thickness tears (42.6%). Surgical technique and adjunctive domains demonstrated low concordance, including arthroscopic versus open repair (3.7% concordant; 21.3% discordant) and biologic therapies (3.7% concordant; 16.7% discordant). In multivariable analysis, longer video duration was associated with higher concordance (odds ratio 1.14 per minute [95% confidence interval: 1.02-1.27]; *P* = .021). Higher view count was associated with lower concordance (odds ratio 0.63 per log_10_(views+1) [95% confidence interval: 0.43-0.91]; *P* = .014). The uploader category was not significantly associated with concordance.

**Conclusion:**

YouTube videos addressing rotator cuff tear management demonstrate low concordance with the 2025 AAOS CPG, with discordance concentrated in surgical approach and adjunctive therapy domains. Higher view counts were associated with lower guideline concordance.

Shoulder pain accounts for an estimated 4.5 million outpatient clinic visits annually in the United States.^19^ Rotator cuff pathology is the most common underlying diagnosis and arises from both acute traumatic injury and chronic degenerative processes.^9^^,^^10^ Rotator cuff tears are associated with pain, loss of strength, functional limitation, and reduced health-related quality of life, underscoring the importance of optimizing management strategies.^7^^,^^18^^,^^24^^,^^25^

In August 2025, the American Academy of Orthopaedic Surgeons (AAOS) released an updated evidence-based Clinical Practice Guideline (CPG) for the management of rotator cuff injuries.^1^ The AAOS CPG is widely used to guide operative and non-operative care and emphasizes patient selection and surgical decision-making, including indications for repair and timing of intervention. For traumatic full-thickness tears, early arthroscopic repair is recommended to optimize tendon healing and functional recovery.^6^^,^^17^^,^^21^ In addition, arthroscopic rotator cuff repair remains a reasonable and effective option in selected patients older than 65 years, challenging the notion that age alone should preclude surgery.^2^^,^^15^^,^^26^ The guideline also provides evidence-based recommendations across perioperative decision points such as operative versus non-operative management, concomitant procedures (eg, acromioplasty), and adjunctive therapies (eg, corticosteroid injections), which inform patient counseling and surgical planning.

Concurrently, many patients seek musculoskeletal health information online, with YouTube representing a common and highly accessible platform.^5^^,^^20^^,^^28^ However, YouTube content is not subject to formal peer review, and prior studies across orthopedic subspecialties have demonstrated that videos frequently contain complete or inaccurate information.^3^^,^^4^^,^^16^ Although prior studies have evaluated the general quality of YouTube as a source of patient education, the extent to which YouTube content addressing indications, timing, and surgical decision-making for arthroscopic rotator cuff repair aligns with contemporary AAOS CPG recommendations remains unknown.

Therefore, the purposes of this study were to (1) evaluate concordance of YouTube videos discussing indications and surgical timing information for arthroscopic rotator cuff repair with the AAOS CPG, (2) identify guideline domains in which recommendations are discordant or not addressed, and (3) determine whether video characteristics are associated with guideline concordance. We hypothesized that widely viewed videos would demonstrate low concordance with AAOS CPG recommendations, that operative recommendations would be more frequently discussed than non-operative management options, and that higher-view counts would correlate with lower concordance.

## Methods

### Study design and reporting framework

This study was a cross-sectional content analysis of publicly available YouTube videos evaluating concordance of video-based patient education with the 2025 AAOS CPG for management of rotator cuff injuries. The study was designed to assess video content discussing surgical indications, timing, and decision-making relevant to arthroscopic rotator cuff repair. Given the use of publicly available content without human subjects' interaction or identifiable private information, institutional review board oversight was not required.

### Search strategy and video identification

A structured YouTube search was performed between January 14, 2026, and January 17, 2026, using predefined, patient-centered search terms intended to capture decision-making content related to rotator cuff tear management and rotator cuff repair (eg, “rotator cuff tear treatment,” “do I need surgery for rotator cuff tear,” “rotator cuff surgery vs. physical therapy,” and related terms). These terms were selected a priori to reflect common, lay-language queries that patients are likely to enter when seeking guidance about rotator cuff tear management, rather than clinical terminology, an approach consistent with the search strategies employed in prior YouTube content analyses in orthopedics.^14^^,^^16^^,^^22^^,^^27^ Searches were conducted in a standardized manner using Google Chrome (Version 135.0.7049.96) in incognito mode to minimize personalization of results. The following filters and settings were standardized across all searches: the default “Relevance” sorting option was used, no additional search filters (eg, upload date, type, and duration) were applied, and Restricted Mode was turned off. The location was manually set to “United States” using the settings accessible via the browser's YouTube interface. For each search term, the first 50 results were screened, consistent with prior methodologies evaluating online medical content and reflecting typical user engagement with early search results.^13^^,^^14^

For each video, the URL was recorded at the time of screening. Duplicate videos identified across search terms were removed prior to full screening. A screening log was maintained to document the number of videos identified, duplicates removed, and exclusions at each stage.

### Eligibility criteria

Videos were eligible for inclusion if they were ≥1 minute (to exclude short-form “YouTube Shorts” and similarly brief content unlikely to provide decision-making guidance) and English-language content that discussed rotator cuff tears and/or rotator cuff repair with educational intent related to management or treatment (operative and/or non-operative). Videos were excluded for any of the following: (1) purely post-operative rehabilitation exercise demonstrations without discussion of management decisions (eg exercise-only protocols), (2) non-educational advertisements or marketing content without substantive educational discussion, and (3) content unrelated to human rotator cuff pathology (eg, veterinary, non-medical, or unrelated shoulder conditions).

### Data extraction and video characteristics

For each included video, the following characteristics were extracted directly from the YouTube interface on the date of review: view count, video duration (minutes), upload date, and uploader category, which was predefined as company, physician, or academic institution based on the uploader identity and channel description.

Channels affiliated with hospitals, universities, academic departments, or residency/fellowship programs were coded as Academic Institution. Channels clearly representing an individual physician or physician group providing clinical education were coded as Physician. All remaining organizational channels, commercial entities, clinics, and non-academic brands were coded as company. When uploader type was ambiguous, categorization was determined by consensus after review of channel metadata.

### American Academy of Orthopaedic Surgeons guideline selection and operationalization

To evaluate concordance, AAOS CPG statements were operationalized into a rubric focused on indications and decision-making relevant to rotator cuff pathology and arthroscopic rotator cuff repair. Guideline statements were translated into discrete, codable items that could be assessed in patient-facing video content. The final rubric included 9 items spanning domains of (1) non-operative management for small-medium full-thickness tears, (2) indications for surgical repair in full-thickness tears, (3) high-grade partial-thickness tears, (4) role of acromioplasty, (5) arthroscopic vs. open repair, (6) single-row vs. double-row constructs, (7) corticosteroid injections, (8) biologics and augmentation, and (9) massive/irreparable tear management.

Each rubric item was coded using a 3-level scheme. Concordant signified the video's recommendation or statement accurately reflected the AAOS CPG recommendation for that item. Discordant referred to the video's recommendation contradicting or misrepresenting the AAOS CPG recommendation for that item. Finally, not addressed was selected when the video did not discuss the item. Importantly, omission was captured as “not addressed” and analyzed separately from discordant content.

### Concordance scoring

For each video, an overall guideline concordance percentage was calculated as: (number of concordant items ÷ total rubric items [9]) x 100%.

### Rater training, independent review, and consensus process

Two raters (S.T. and M.J.) independently reviewed all included videos and completed item-level coding for the 9-item rubric as well as extraction of video characteristics. Prior to formal coding, raters completed standardized training that included (1) review of the AAOS CPG statements used for rubric construction; (2) review of item definitions and examples of concordant, discordant, and not addressed classifications; and (3) pilot coding of 5 videos with discussion to refine application of the rubric.

After independent coding, raters held a structured consensus meeting to reconcile discrepancies item-by-item. The dataset used for analysis reflects the post-consensus coding and final agreed-upon values for each video. Interrater reliability was calculated on the pre-consensus ratings using Cohen's k and is reported as k = 0.84.

### Outcomes

The primary outcome was overall per-video AAOS guideline concordance percentage. Secondary outcomes included item-level distributions of concordant, discordant, and not addressed classifications for each rubric item, as well as associations between guideline concordance and video characteristics (view count, duration, upload year, and uploader category).

### Statistical analysis

Continuous variables were summarized as mean ± standard deviation or median (interquartile range [IQR]) based on distribution. Categorical variables were summarized as counts and percentages. A comprehensive concordance was categorized using an a priori ordinal scale defined by thirds of the 9-item rubric: low (<33.3%; 0-2 domains concordant), moderate (33.3-66.6%; 3-5 domains), and high (≥66.7%; ≥6 domains). Univariable and multivariable logistic regression models were constructed with above-median concordance as the dependent variable. Prespecified covariates included view count, video duration (minutes), upload year, and uploader category (Academic Institution as reference). Model results were reported as odds ratios (ORs) with 95% confidence intervals (CIs). Statistical significance was set at *P* = .05. Analyses were performed using Stata version 18 (StataCorp, College Station, TX, USA).

## Results

### Study selection

A total of 257 videos were identified through the predefined YouTube search strategy. After removal of 46 duplicate videos, 211 videos underwent title and description screening. Of these, 92 videos were excluded based on predefined criteria, most commonly for exclusively post-operative rehabilitation content (n = 51), non-educational advertisements (n = 24), or non-rotator cuff-related content (n = 17). Eleven videos were further deemed unsuitable for analysis due to foreign language, poor audio quality, and being a YouTube short. The final analytic sample consisted of 108 videos, which were included for guideline concordance analysis (Fig. 1).

**Figure 1:**
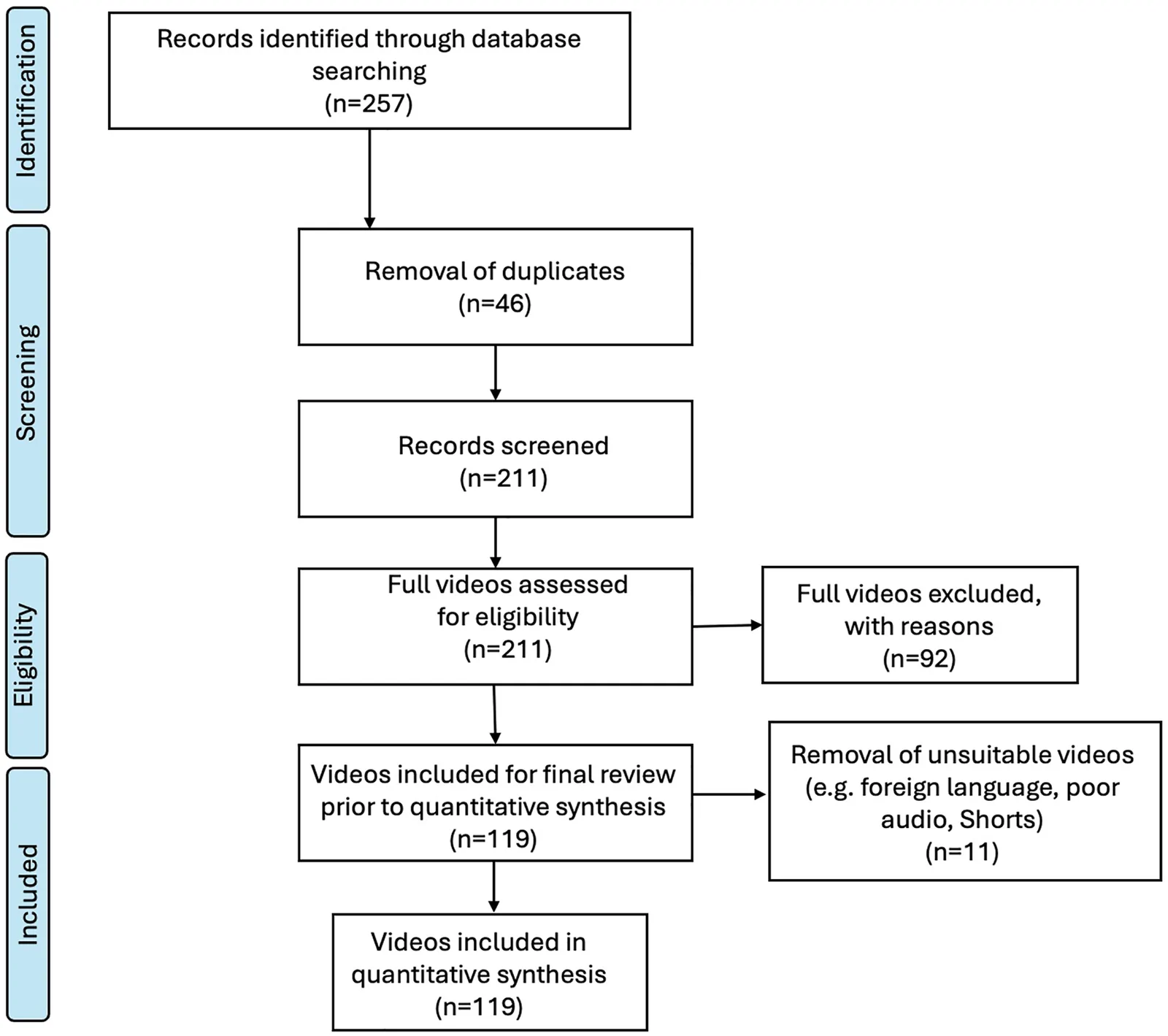
A flowchart of the videos screening performed in this study.

### Video characteristics

The 108 videos had a median upload year of 2021 (IQR, 2018-2023). Median video duration was 2.8 minutes (IQR, 1.9-6.4). View count was right-skewed, with a median count of 2,782 views (IQR, 502-20,574) (Table I).

**Table I:** Video characteristics overall and by uploader category.

Characteristic	Overall (n = 108)	Company (n = 74)	Physician (n = 21)	Academic institution (n = 13)
Upload yr, median (IQR)	2021 (2018-2023)	2021 (2019-2023)	2021 (2018-2023)	2020 (2018-2022)
Duration, min, median (IQR)	2.8 (1.9-6.4)	3.5 (2.0-6.5)	2.3 (2.0-5.1)	2.0 (1.0-3.8)
Views, median (IQR)	2,782 (502-20,574)	2,232 (455-12,728)	4,639 (671-66,923)	9,412 (1,095-63,338)
Guideline concordance (%), median (IQR)	11.1 (0.0-33.3)	11.1 (0.0-33.3)	22.2 (0.0-44.4)	33.3 (0.0-44.4)

*IQR*, interquartile range.

The uploader category mostly consisted of company sources (75/108, 68.5%), followed by physicians (21/108, 19.4%) and academic institutions (13/108, 12.0%).

### Overall American Academy of Orthopaedic Surgeons guideline concordance

Across the 9 AAOS guideline-derived rubric items, the median per-video guideline concordance was 11.1% (IQR, 0.0-33.3), corresponding to a median of 1 concordant item out of 9. On the a priori ordinal scale, 68 videos (63.0%) demonstrated low comprehensive concordance (<33.3% of domains concordant), 38 (35.2%) moderate (33.3-66.6%), and 2 (1.9%) high (≥66.7%) (Table II). The mean number of guideline items coded as “not addressed” per video was 6.4 ± 1.9. The highest observed concordance was 66.7% (6/9 items concordant). A concordance of 0% was observed in 44 videos. Of the 396 item-level codings, 42 (10.6%) were discordant and 353 (89.1%) were not addressed. There was a mean of 8.0 ± 1.3 of 9 items unaddressed per video. Overall guideline concordance was 18.3% with an absence of concordance in 81.7% (10.5% discordant, 71.1% not addressed) (Fig. 2). The lower per-video median of 11.1% reflects the right-skewed distribution of concordant items across videos.

**Table II:** A priori ordinal scale for measurement of high and low concordance.

Grade	Definition	n (%)
Low	<33.3% (0-2 domains of 9 concordant)	68 (63.0%)
Moderate	33.3-66.6% (3-5 domains)	38 (35.2%)
High	≥66.7% (≥6 domains)	2 (1.9%)

**Figure 2:**
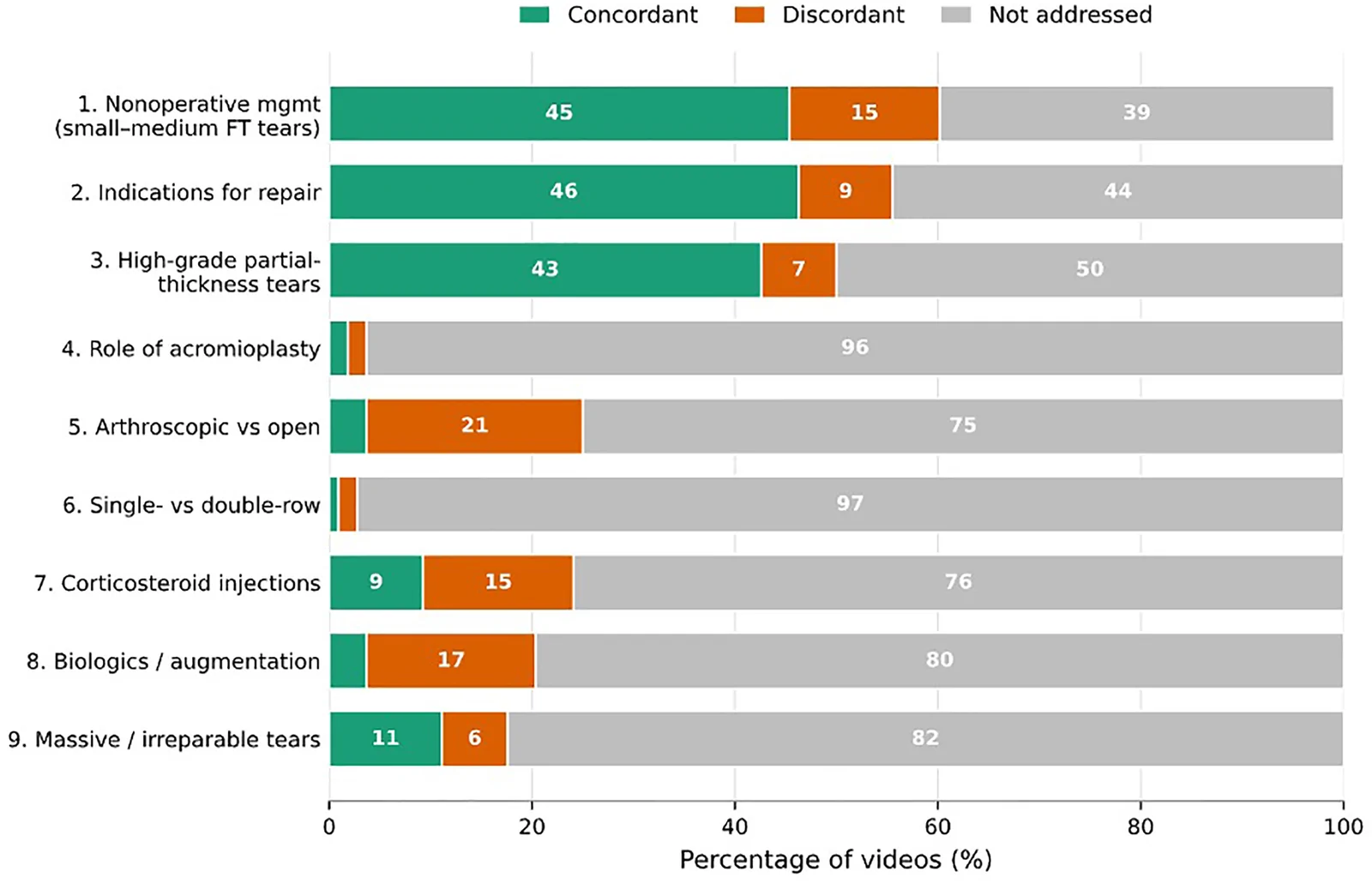
Distribution of concordance classifications across the 9 AAOS guideline-derived rubric items (n = 108 videos). Stacked bars show, for each item, the percentage of videos coded as concordant, discordant, or not addressed. *AAOS*, American Academy of Orthopaedic Surgeons.

### Concordance by American Academy of Orthopaedic Surgeons guideline item

#### Operative vs. non-operative management

Accurate acknowledgment that physical therapy may result in meaningful patient-reported outcome improvement for symptomatic small- to medium-sized full-thickness rotator cuff tears was observed in 49/108 (45.4%) videos (Item 1). Discordant statements were present in 16/108 (14.8%), and 42/108 (38.9%) videos did not address this recommendation (Table III).

**Table III:** Concordance with AAOS guideline-based rubric items (n = 108).

AAOS rubric item	Concordant, n (%)	Discordant, n (%)	Not addressed, n (%)
Item 1. Non-operative management for small-medium full-thickness tears	49 (45.4%)	16 (14.8%)	42 (38.9%)
Item 2. Indications for surgical repair in full-thickness tears	50 (46.3%)	10 (9.3%)	48 (44.4%)
Item 3. High-grade partial-thickness tears (after failed conservative tx)	46 (42.6%)	8 (7.4%)	54 (50.0%)
Item 4. Role of acromioplasty	2 (1.9%)	2 (1.9%)	104 (96.3%)
Item 5. Arthroscopic vs. open repair	4 (3.7%)	23 (21.3%)	81 (75.0%)
Item 6. Single-row vs. double-row constructs	1 (0.9%)	2 (1.9%)	105 (97.2%)
Item 7. Corticosteroid injections	10 (9.3%)	16 (14.8%)	82 (75.9%)
Item 8. Biologics and augmentation	4 (3.7%)	18 (16.7%)	86 (79.6%)
Item 9. Massive/irreparable tear management	12 (11.1%)	7 (6.5%)	89 (82.4%)

*AAOS*, American Academy of Orthopaedic Surgeons.

Regarding indications for surgical repair, 50/108 (46.3%) videos accurately stated that healed rotator cuff repairs are associated with superior functional outcomes compared with unhealed repairs or non-operative management (Item 2). Discordant content was present in 10/108 (9.3%), while 48/108 (44.4%) did not address surgical indications.

#### Partial thickness rotator cuff tears

Management of high-grade partial-thickness rotator cuff tears following failure of conservative treatment was accurately described in 46/108 (42.6%) videos (Item 3). Discordant recommendations were identified in 8/108 (7.4%), and 54/108 (50.0%) videos did not address this topic.

#### Surgical technique and approach

The role of concomitant acromioplasty was addressed in 4/108 (3.7%) videos, with 2/108 (1.9%) accurately reflecting AAOS recommendations and 2/108 (1.9%) providing discordant information; 104/108 (96.3%) videos did not address acromioplasty (Item 4).

Comparison between arthroscopic and open rotator cuff repair was discussed in 27/108 (25.0%) videos (Item 5). Of these, 4/108 (3.7%) were concordant with AAOS recommendations, 23/108 (21.3%) were discordant, and 81/108 (75.0%) did not address surgical approach.

Discussion of single-row versus double-row repair constructs occurred in 3/108 (2.8%) videos (Item 6), with 1/108 (0.9%) concordant and 2/108 (1.9%) discordant; 105/108 (97.2%) did not address repair construct selection.

#### Adjunctive therapies and injections

Corticosteroid injection use was addressed in 26/108 (24.1%) videos (Item 7). Accurate representation of AAOS guidance occurred in 10/108 (9.3%), discordant content in 16/108 (14.8%), and 82/108 (75.9%) did not address injections.

Biologic therapies and augmentation strategies were discussed in 22/108 (20.4%) videos (Item 8). Concordant information was present in 4/108 (3.7%), discordant information in 18/108 (16.7%), and 86/108 (79.6%) did not address biologic adjuncts.

#### Management of massive or irreparable tears

Management strategies for massive or irreparable rotator cuff tears were addressed in 19/108 (17.6%) videos (Item 9). Of these, 12/108 (11.1%) accurately reflected AAOS guidance, 7/108 (6.5%) were discordant, and 89/108 (82.4%) did not address this recommendation.

#### Association between video characteristics and guideline concordance

In multivariable logistic regression analysis evaluating factors associated with above-median guideline concordance, higher view count (log_10_[views+1]) was associated with lower odds of above-median concordance (OR, 0.63 [95% CI: 0.43-0.91]; *P* = .014). Longer video duration was associated with higher odds of above-median concordance (OR per additional minute, 1.14 [95% CI: 1.02-1.27]; *P* = .021). Upload year was not associated with guideline concordance. The creator category demonstrated no statistically significant association with concordance in adjusted analysis (Table IV).

**Table IV:** Factors associated with above-median guideline concordance.

Predictor	OR (95% CI)	*P* value
log_10_(views+1)	**0.63 (0.43-0.91)**	**.014∗**
Video duration (per 1 min)	**1.14 (1.02-1.27)**	**.021∗**
Upload yr (per 1 yr)	0.96 (0.86-1.07)	.446
Creator: company vs. academic institution	0.31 (0.08-1.20)	.091
Creator: physician vs. academic institution	0.66 (0.14-3.06)	.594

*CI*, confidence interval; *OR*, odds ratio.

*P < .05 are indicated in bold.

## Discussion

In this cross-sectional analysis of 108 YouTube videos addressing rotator cuff tear management, overall concordance with the 2025 AAOS CPG was low, with a median concordance of 11.1% (1 of 9 decision points). A mean of 6.4 guideline items per video coded as “not addressed.” In multivariable analysis, longer video duration was associated with higher guideline concordance, whereas higher view count was independently associated with lower concordance. Uploader category was not significantly associated with alignment with AAOS recommendations.

The highest concordance rates were observed for foundational treatment concepts, such as acknowledgment that physical therapy can improve patient-reported outcomes in small-to-medium full-thickness tears (45.4%), discussion of surgical indications (46.3%), and escalation pathways for high-grade partial-thickness tears (42.6%). However, substantial proportions of videos failed to address these concepts altogether; 38.9% omitted discussion of physical therapy effectiveness and 44.4% omitted surgical indications. Even among commonly discussed domains, omission was frequent, potentially limiting patients' understanding of the full spectrum of management options.

Operative technique and adjunct domains demonstrated particularly low concordance, and the discordance concentrated in these domains is arguably the more consequential finding for patient care. When the surgical approach was addressed, discordant superiority claims occurred in 21.3% of videos despite guideline statements supporting equivalent long-term outcomes between open and arthroscopic repair. Framed against the 27 videos that actually discussed the topic, 23 (85.2%) conveyed discordant information. Adjunctive therapies followed the same pattern. Of the 22 videos that discussed biologic augmentation, 18 (81.8%) were discordant, as were 16 of the 26 videos (61.5%) that addressed corticosteroid injections. Thus, although discordance was uncommon as a fraction of the full sample, it was prominent in the subset of videos that elected to discuss these topics. In other words, when a video addressed surgical techniques or adjuncts, it was more likely to mislead than to inform. Because these domains often influence patient expectations prior to consultation, inconsistent framing may disproportionately affect shared decision-making. By inconsistent framing we mean that, beyond overstating the certainty of preference-sensitive options, the corpus of videos delivered directly contradictory guidance on certain decision points, such that a patient sampling several videos would encounter opposing recommendations. This was clearest for topics such as corticosteroid injections, where 10 videos conveyed a concordant message that repeated injections may compromise tendon integrity, while 16 promoted repeated injections without that caution. Because patients rarely rely on a single source, this aggregate inconsistency may undermine calibrated expectations more than any individual discordant claim.

Discordant content clustered into 3 recurring categories. The first, overstatement of surgical superiority, comprised claims favoring one operative approach despite guideline-supported equivalence (eg, 21.3% discordant for arthroscopic versus open repair). Less commonly, there were discordant discussions surrounding single- versus double-row constructs (1.9%) and concomitant acromioplasty (1.9%). The second category, overpromotion of adjunctive and biologic therapies, consisted of biologic augmentation (16.7%) and corticosteroid injections (14.8%) and was presented with greater certainty of benefit than the current evidence supports. The third, misrepresentation of non-operative management and operative indications, included discordant framing of physical therapy effectiveness (14.8%), indications for repair (9.3%), escalation for high-grade partial-thickness tears (7.4%), and management of massive or irreparable tears (6.5%). Discordance was therefore concentrated in the 2 domains most susceptible to promotional or preference-driven messaging rather than in foundational counseling about the role of non-operative care. Given that company-affiliated channels produced the majority of videos analyzed (68.5%), this distribution warrants particular caution because these are precisely the areas in which unbalanced messaging may shape patient expectations before any clinical encounter.

These findings align with prior evaluations of YouTube as a source of orthopedic patient education, including content related to the shoulder. Springer et al^22^ reported poor informational quality, accuracy, and reliability among YouTube videos addressing post-operative rehabilitation following rotator cuff repair, and Kuenze et al^12^ similarly described low quality for general rotator cuff disease information. Our study extends this literature by anchoring analysis to the updated 2025 AAOS CPG and focusing specifically on decision-making domains relevant to arthroscopic rotator cuff repair rather than post-operative exercise content. In addition, the high frequency of “not addressed” coding (mean 6.4 items per video) is consistent with the broader pattern of incomplete online treatment information reported in other conditions, such as knee osteoarthritis.^27^ Taken together, these studies and ours suggest that incompleteness may be the predominant limitation of online videos even more than overt inaccuracy, reinforcing the importance of distinguishing omission from discordance in guideline-based evaluation.

The inverse association between view count and guideline concordance mirrors observations in other orthopedic YouTube analyses. Staunton et al^23^ reported that higher educational quality was associated with fewer views in scoliosis-related videos, and Jones et al^8^ found that “useful” Dupuytren's disease videos had lower mean view counts. While causal inference is not possible in the present study, these data suggest that engagement metrics such as views may not reflect educational alignment with evidence-based recommendations and may instead reflect factors such as production style, brevity, or messaging simplicity. The positive association between video duration and concordance supports this interpretation, as longer videos may provide greater opportunity to address multiple decision points and provide balanced framing, whereas shorter videos may omit nuance to maximize accessibility.

The uploader category was not independently associated with concordance. Notably, company sources represented the majority (68.5%) of included videos. Although this study did not evaluate financial disclosures or intent, the predominance of commercially affiliated content underscores the importance of careful interpretation of online educational material, particularly in areas such as adjunctive devices and biologics where the evidence base continues to evolve.

For practicing arthroscopists, these findings suggest that patients may frequently arrive with partial or selectively framed information regarding rotator cuff tear management. Proactive discussion of expected outcomes with physical therapy, surgical indications, escalation pathways for partial-thickness tears, and the conditional role of adjunctive therapies may help align expectations and reinforce evidence-based decision-making.^11^

### Limitations

This study has limitations. First, YouTube search results and engagement metrics are dynamic and may vary by time, location, and personalization; our findings reflect a snapshot during the study period. Secondly, views, likes, and comment counts are imperfect surrogates for information exposure and do not capture watch time or viewer comprehension.

Guideline-based rubrics necessarily simplify complex decision-making; some guideline topics may be omitted because videos target a general audience or focus on a single aspect of care. Additionally, the uploader classification may be imperfect, particularly when channel affiliations are unclear.

Furthermore, the median upload year of 2021 indicates that many videos predate the 2025 guideline update; however, several core recommendations evaluated in this study (eg, physical therapy as a reasonable initial strategy in select tears and long-term equivalence of open and arthroscopic repairs) have remained stable across iterations.

Finally, this analysis was restricted to English-language YouTube content and may not generalize to other platforms or languages.

## Conclusion

YouTube videos addressing rotator cuff tear management demonstrate low concordance with the 2025 AAOS CPG, with discordance concentrated in surgical approach and adjunctive therapy domains. Higher view counts were associated with lower guideline concordance, but primarily due to incomplete coverage; a narrowly scoped video is not inherently a poor resource. Patients should be wary of reliance on YouTube videos for information regarding rotator cuff pathology and surgery. Similarly, surgeons should be aware of potential patient exposure to incomplete information regarding the management of rotator cuff injuries. Accordingly, surgeons should provide comprehensive information to their patients when deciding on a treatment plan.

## Disclaimers:

Funding: No funding was disclosed by the authors.

Conflicts of interest: The authors, their immediate families, and any research foundations with which they are affiliated have not received any financial payments or other benefits from any commercial entity related to the subject of this article.
